# 8-[(1*E*)-1-(2-Aminophenyl­iminio)eth­yl]-2-oxo-2*H*-chromen-7-olate

**DOI:** 10.1107/S1600536810023093

**Published:** 2010-06-26

**Authors:** E. S. Aazam, A. F. El Husseiny, H. M. Al-Amri, Orhan Büyükgüngör

**Affiliations:** a Department of Chemistry, Girls Section, University of King Abdulaziz, PO Box 6171, Jeddah 21442, Saudi Arabia; b Department of Chemistry, Faculty of Science, Alexandria University, Egypt; c Department of Physics, Ondokuz Mayıs University, TR-55139 Samsun, Turkey

## Abstract

The title Schiff base, C_17_H_14_N_2_O_3_, exists as an NH tautomer with the H atom of the phenol group transferred to the imine N atom. The iminium H atom is involved in a strong intra­molecular N^+^—H⋯O^−^ hydrogen bond to the phenolate O atom, forming an *S*(6) motif. In the crystal structure, N—H⋯O hydrogen bonds form a *C*(9) chain parallel to [100] and a *C*(11) chain parallel to [010], while C—H⋯O hydrogen bonds form a *C*(11) chain parallel to [010]. The combination of N—H⋯O and C—H⋯O hydrogen bonds generates *R*_4_^3^(30) rings parallel to the *ab* plane

## Related literature

For related structures, see: Patil *et al.* (2010 ▶); Aazam *et al.* (2006 ▶); Filarowski (2005 ▶); El Husseiny *et al.* (2008 ▶); Aazam *et al.* (2008 ▶); Karabıyık *et al.* (2008 ▶). For the graph-set analysis of hydrogen-bond patterns, see: Bernstein *et al.* (1995 ▶). 
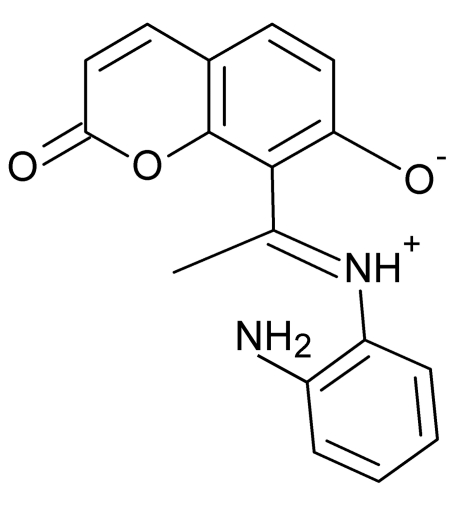


## Experimental

### 

#### Crystal data

C_17_H_14_N_2_O_3_
*M* *_r_* = 294.30
Orthorhombic, 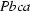

*a* = 7.5462 (3) Å
*b* = 18.9324 (11) Å
*c* = 20.0445 (9) Å
*V* = 2863.7 (2) Å^3^
*Z* = 8
Mo *K*α radiation
μ = 0.10 mm^−1^
*T* = 296 K
0.44 × 0.29 × 0.20 mm

#### Data collection

Stoe IPDS 2 diffractometer
Absorption correction: integration (*X-RED32*; Stoe & Cie, 2002 ▶) *T*_min_ = 0.962, *T*_max_ = 0.984
25561 measured reflections
2868 independent reflections
1972 reflections with *I* > 2σ(*I*)
*R*_int_ = 0.039

#### Refinement

*R*[*F* ^2^ > 2σ(*F* ^2^)] = 0.038
*wR*(*F* ^2^) = 0.095
*S* = 1.00
2868 reflections
212 parameters
1 restraint
H atoms treated by a mixture of independent and constrained refinement
Δρ_max_ = 0.11 e Å^−3^
Δρ_min_ = −0.15 e Å^−3^

### 

Data collection: *X-AREA* (Stoe & Cie, 2002 ▶); cell refinement: *X-AREA*; data reduction: *X-RED32* (Stoe & Cie, 2002 ▶); program(s) used to solve structure: *SHELXS97* (Sheldrick, 2008 ▶); program(s) used to refine structure: *SHELXL97* (Sheldrick, 2008 ▶); molecular graphics: *ORTEP-3 for Windows* (Farrugia, 1997 ▶); software used to prepare material for publication: *WinGX* (Farrugia, 1999 ▶).

## Supplementary Material

Crystal structure: contains datablocks I, global. DOI: 10.1107/S1600536810023093/gk2282sup1.cif

Structure factors: contains datablocks I. DOI: 10.1107/S1600536810023093/gk2282Isup2.hkl

Additional supplementary materials:  crystallographic information; 3D view; checkCIF report

## Figures and Tables

**Table 1:** Hydrogen-bond geometry (Å, °)

*D*—H⋯*A*	*D*—H	H⋯*A*	*D*⋯*A*	*D*—H⋯*A*
N1—H1⋯O1	0.95 (2)	1.56 (2)	2.4534 (17)	155 (2)
N2—H2*A*⋯O1^i^	0.92 (2)	2.13 (2)	2.9933 (19)	154.7 (15)
N2—H2*B*⋯O2^ii^	0.91 (2)	2.37 (2)	3.1203 (19)	138.7 (17)
C5—H5⋯O2^iii^	0.93	2.48	3.242 (2)	139

Symmetry codes: (i) 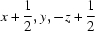; (ii) 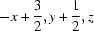; (iii) 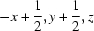.

**Fig. 1.**
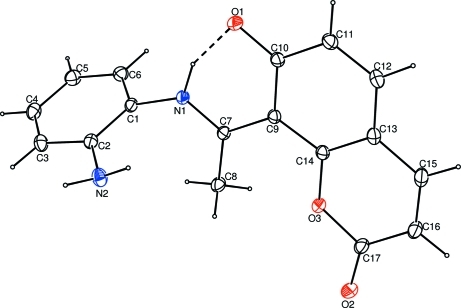
A view of one molecule of (I), showing the atom-numbering scheme. Displacement ellipsoids are drawn at the 30% probability level. Hydrogen bonds are indicated by dashed lines.

**Fig. 2.**
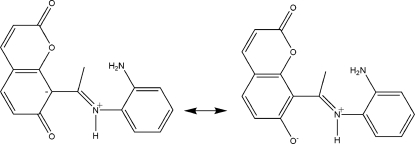
Zwitterionic canonical forms of I.

**Fig. 3.**
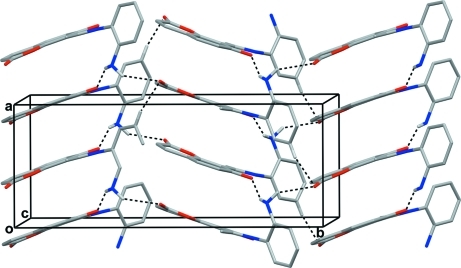
Part of the crystal structure of (I), showing the formation of a hydrogen-bonded sheet built from C(9) and C(11) chains. For the sake of clarity, H atoms not involved in the motif shown have been omitted.

## supplementary crystallographic information

### Comment

Coumarin and its derivatives have been a subject of numerous investigations due to their diverse biological activities, interesting photophysical, photochemical and metal binding properties. Schiff base complexes play an important role in coordination chemistry (Patil *et al.*, 2010). We have recently reported synthesis, single-crystal X-ray diffraction studies, characterization and antibacterial activity of a Schiff-base ligand incorporating coumarin moiety and their metal complexes (Aazam *et al.*, 2006; El Husseiny, *et al.*, 2008; Aazam *et al.*, 2008). In this work, we would like to present an X-ray investigation of a newly synthesized coumarin Schiff base derived from 8-acetyl-7-hydroxycoumarin and *o*-phenylenediamine. We report here the crystal structure of (I) (Figure 1).

Schiff bases exhibit two well known tautomeric forms *viz*. OH and NH tautomers. NH tautomers can be regarded as a resonance hybrid of two canonical structures the non-charged quinoid and ionic zwitterionic forms. Our investigations show that compound (I) formula can be given as a combination of two canonical forms: phenolate with negative charge predominantly at the phenolate O atom or as chromen-8-ide with negative charge at the C atom. Compound I is a hyprid of two zwitterionic canonical forms (Fig. 2) having N^+^—H bond (0.952 Å) longer than standared interatomic separations observed in neutral N—H bonds (0.878 Å)(Karabıyık *et al.*, 2008). Compound (I) crystallizes in the orthorhombic space group Pbca (No. 61) with one molecule per asymmetric unit. The iminium H atom is almost coplanar with the coumarin ring (deviation from the coumarin plane 0.0444 Å) and on the same side of the molecule as the phenolic O atom, allowing the formation of an intramolecular N—H···O hydrogen bond. A summary of bond lengths and angles of the ketoimine system is presented in Table 1.

In order to compare the bond lengths found in (I) with other molecules containing similar functional groups, a comparison with a previous search in the Cambridge Structural Database (CSD) was performed (Filarowski, 2005). The comparison with the mean bond lengths of similar molecules clearly tends to confirm that (I) is *cis*-ketoimine tauutomer and that the C7—N1 in agreement with double-bond character, the C10—O1 is 1.3040 Å, this bond is not double (keto group) and is one of the longest among similar iminium derivatives, also C7—C9 bond length is not a typical single-bond. Moreover C9—C14 ring is in agreement with the cyclohexadienide bond character. The intramolecular hydrogen bond produces S(6) motif (Bernstein *et al.*, 1995). The amino atom N2 in the molecule at (*x*, *y*, *z*) acts as a hydrogen-bond donor (Table 1) to atom O1^i^ so forming a C(9) chain running parallel to the [100] direction. Amino atom N2 in the molecule at (*x*, *y*, *z*) acts as a hydrogen-bond donor to atom O2^ii^ so forming a C(11) chain running parallel to the [010] direction. Similarly, atom C5 in the molecule at (*x*, *y*, *z*) acts as a hydrogen-bond donor to atom O2^iii^ so forming a C(11) chain running parallel to the [010] direction. The combination of N—H···O and C—H···O hydrogen bonds generates *R*_4_^3^(30) rings parallel to the *ab* plane (Fig. 3).

### Experimental

A clear solution of *o*-phenylenediamine (0.26 g, 2.5 mmol) in 10 ml of ethanol was added to a warm solution of 8-acetyl-7-hydroxy coumarin (0.5 g, 2.5 mmol) in the same solvent (30 ml). The resulting mixture was refluxed for 2–3 h. The yellow product was precipitated, filtered off and washed with ethanol followed by diethyl ether, dried in a vacuum desiccator and crystallized from chloroform/ethanol (2:1). Single crystals were obtained by slow evaporation of dichloromethane solution of I at room temperature; Yield (75%), m.p. 480 K. Purity of the ligand was checked using TLC; (methanol: benzene, 1:4).

### Refinement

All H atoms bound to C atoms were refined using a riding model, with C—H = 0.93Å and *U*_iso_(H) = 1.2*U*_eq_(C) for aromatic C atoms and C—H = 0.96Å and *U*_iso_(H) = 1.5*U*_eq_(C) for methyl C atoms. Amino H atoms bound to N atom were located in difference maps and refined freely. Other H atom bound to N atom was located in difference maps and refined subject to a *DFIX* restraint of N—H = 0.86 (2) Å.

### Crystal data

**Table:**

C_17_H_14_N_2_O_3_	*F*(000) = 1232
*M**_r_* = 294.30	*D*_x_ = 1.365 Mg m^−^^3^
Orthorhombic, *P**b**c**a*	Mo *K*α radiation, λ = 0.71073 Å
Hall symbol: -P 2ac 2ab	Cell parameters from 25561 reflections
*a* = 7.5462 (3) Å	θ = 2.0–26.7°
*b* = 18.9324 (11) Å	µ = 0.10 mm^−^^1^
*c* = 20.0445 (9) Å	*T* = 296 K
*V* = 2863.7 (2) Å^3^	Prism, yellow
*Z* = 8	0.44 × 0.29 × 0.20 mm

### Data collection

**Table:**

Stoe IPDS 2 diffractometer	2868 independent reflections
Radiation source: fine-focus sealed tube	1972 reflections with *I* > 2σ(*I*)
graphite	*R*_int_ = 0.039
rotation method scans	θ_max_ = 26.2°, θ_min_ = 2.0°
Absorption correction: integration (*X-RED32*; Stoe & Cie, 2002)	*h* = −9→9
*T*_min_ = 0.962, *T*_max_ = 0.984	*k* = −23→23
25561 measured reflections	*l* = −24→24

### Refinement

**Table:**

Refinement on *F*^2^	Primary atom site location: structure-invariant direct methods
Least-squares matrix: full	Secondary atom site location: difference Fourier map
*R*[*F*^2^ > 2σ(*F*^2^)] = 0.038	Hydrogen site location: inferred from neighbouring sites
*wR*(*F*^2^) = 0.095	H atoms treated by a mixture of independent and constrained refinement
*S* = 1.00	*w* = 1/[σ^2^(*F*_o_^2^) + (0.0515*P*)^2^ + ] where *P* = (*F*_o_^2^ + 2*F*_c_^2^)/3
2868 reflections	(Δ/σ)_max_ < 0.001
212 parameters	Δρ_max_ = 0.11 e Å^−^^3^
1 restraint	Δρ_min_ = −0.15 e Å^−^^3^

### Special details

**Table:**

Geometry. All e.s.d.'s (except the e.s.d. in the dihedral angle between two l.s. planes) are estimated using the full covariance matrix. The cell e.s.d.'s are taken into account individually in the estimation of e.s.d.'s in distances, angles and torsion angles; correlations between e.s.d.'s in cell parameters are only used when they are defined by crystal symmetry. An approximate (isotropic) treatment of cell e.s.d.'s is used for estimating e.s.d.'s involving l.s. planes.
Refinement. Refinement of *F*^2^ against ALL reflections. The weighted *R*-factor *wR* and goodness of fit *S* are based on *F*^2^, conventional *R*-factors *R* are based on *F*, with *F* set to zero for negative *F*^2^. The threshold expression of *F*^2^ > σ(*F*^2^) is used only for calculating *R*-factors(gt) *etc*. and is not relevant to the choice of reflections for refinement. *R*-factors based on *F*^2^ are statistically about twice as large as those based on *F*, and *R*- factors based on ALL data will be even larger.

### Fractional atomic coordinates and isotropic or equivalent isotropic displacement parameters (Å^2^)

**Table:**

	*x*	*y*	*z*	*U*_iso_*/*U*_eq_	
C1	0.3733 (2)	0.81253 (7)	0.38471 (7)	0.0462 (4)	
C2	0.5193 (2)	0.83854 (7)	0.41971 (8)	0.0487 (4)	
C3	0.4876 (2)	0.89106 (8)	0.46724 (8)	0.0584 (4)	
H3	0.5822	0.9095	0.4914	0.070*	
C4	0.3197 (3)	0.91605 (8)	0.47909 (9)	0.0632 (5)	
H4	0.3019	0.9507	0.5113	0.076*	
C5	0.1777 (3)	0.89021 (9)	0.44379 (9)	0.0646 (5)	
H5	0.0642	0.9073	0.4519	0.077*	
C6	0.2051 (2)	0.83870 (8)	0.39613 (9)	0.0581 (4)	
H6	0.1098	0.8215	0.3716	0.070*	
C7	0.44310 (18)	0.69702 (7)	0.33766 (7)	0.0446 (3)	
C8	0.4588 (2)	0.66606 (8)	0.40572 (8)	0.0602 (4)	
H8A	0.4121	0.6987	0.4379	0.072*	
H8B	0.5813	0.6569	0.4154	0.072*	
H8C	0.3932	0.6227	0.4077	0.072*	
C9	0.47034 (19)	0.65822 (7)	0.27529 (7)	0.0442 (3)	
C10	0.4368 (2)	0.69219 (7)	0.21251 (8)	0.0491 (4)	
C11	0.4623 (2)	0.65452 (8)	0.15220 (8)	0.0584 (4)	
H11	0.4402	0.6767	0.1117	0.070*	
C12	0.5186 (2)	0.58675 (8)	0.15284 (9)	0.0587 (4)	
H12	0.5332	0.5630	0.1126	0.070*	
C13	0.5555 (2)	0.55134 (7)	0.21273 (8)	0.0525 (4)	
C14	0.53174 (19)	0.58766 (7)	0.27212 (8)	0.0455 (4)	
C15	0.6172 (2)	0.48034 (8)	0.21610 (10)	0.0641 (5)	
H15	0.6307	0.4548	0.1768	0.077*	
C16	0.6561 (2)	0.44943 (8)	0.27412 (10)	0.0671 (5)	
H16	0.6943	0.4027	0.2746	0.081*	
C17	0.6397 (2)	0.48719 (7)	0.33552 (10)	0.0588 (4)	
N1	0.39971 (17)	0.76316 (6)	0.33146 (7)	0.0488 (3)	
N2	0.68956 (19)	0.81302 (8)	0.41013 (8)	0.0631 (4)	
O1	0.38245 (16)	0.75749 (5)	0.20935 (6)	0.0603 (3)	
O2	0.67878 (17)	0.46798 (5)	0.39135 (7)	0.0735 (4)	
O3	0.57044 (14)	0.55503 (5)	0.33133 (5)	0.0541 (3)	
H1	0.390 (3)	0.7742 (10)	0.2853 (8)	0.087 (6)*	
H2A	0.715 (2)	0.7907 (9)	0.3703 (10)	0.071 (5)*	
H2B	0.772 (3)	0.8469 (11)	0.4178 (11)	0.099 (7)*	

### Atomic displacement parameters (Å^2^)

**Table:**

	*U*^11^	*U*^22^	*U*^33^	*U*^12^	*U*^13^	*U*^23^
C1	0.0615 (9)	0.0363 (7)	0.0409 (9)	−0.0009 (6)	0.0025 (7)	0.0007 (6)
C2	0.0586 (9)	0.0435 (7)	0.0439 (9)	−0.0018 (7)	0.0053 (7)	−0.0002 (6)
C3	0.0700 (11)	0.0557 (8)	0.0494 (10)	−0.0061 (8)	0.0042 (8)	−0.0116 (7)
C4	0.0844 (13)	0.0537 (9)	0.0515 (10)	0.0048 (8)	0.0143 (9)	−0.0090 (7)
C5	0.0651 (10)	0.0662 (10)	0.0623 (12)	0.0133 (8)	0.0086 (9)	−0.0029 (9)
C6	0.0586 (10)	0.0577 (9)	0.0579 (11)	0.0039 (7)	−0.0008 (8)	−0.0007 (8)
C7	0.0490 (8)	0.0393 (7)	0.0454 (9)	−0.0045 (6)	−0.0014 (7)	0.0024 (6)
C8	0.0889 (12)	0.0459 (8)	0.0459 (10)	0.0056 (8)	0.0010 (9)	0.0037 (7)
C9	0.0483 (8)	0.0397 (7)	0.0446 (9)	−0.0056 (6)	−0.0026 (6)	−0.0011 (6)
C10	0.0563 (9)	0.0455 (7)	0.0454 (9)	−0.0067 (6)	−0.0048 (7)	0.0001 (6)
C11	0.0734 (11)	0.0588 (9)	0.0431 (10)	−0.0053 (8)	−0.0043 (8)	−0.0011 (7)
C12	0.0681 (10)	0.0594 (9)	0.0486 (11)	−0.0077 (8)	0.0003 (8)	−0.0134 (7)
C13	0.0544 (9)	0.0465 (8)	0.0565 (11)	−0.0054 (6)	−0.0008 (7)	−0.0097 (7)
C14	0.0474 (8)	0.0411 (7)	0.0480 (10)	−0.0076 (6)	−0.0040 (7)	0.0001 (6)
C15	0.0686 (11)	0.0489 (8)	0.0749 (13)	−0.0011 (8)	−0.0002 (10)	−0.0181 (8)
C16	0.0724 (11)	0.0416 (8)	0.0874 (15)	0.0047 (7)	−0.0082 (10)	−0.0091 (9)
C17	0.0586 (10)	0.0384 (7)	0.0795 (14)	−0.0011 (7)	−0.0101 (9)	0.0018 (8)
N1	0.0667 (8)	0.0393 (6)	0.0403 (8)	0.0007 (5)	−0.0026 (6)	−0.0012 (5)
N2	0.0562 (8)	0.0674 (9)	0.0659 (10)	−0.0034 (7)	0.0055 (8)	−0.0179 (8)
O1	0.0862 (8)	0.0476 (6)	0.0472 (7)	0.0064 (5)	−0.0074 (6)	0.0060 (5)
O2	0.0904 (9)	0.0482 (6)	0.0820 (10)	0.0054 (6)	−0.0181 (8)	0.0106 (6)
O3	0.0671 (7)	0.0391 (5)	0.0560 (7)	0.0035 (5)	−0.0068 (5)	0.0008 (5)

### Geometric parameters (Å, °)

**Table:**

C1—C6	1.381 (2)	C9—C10	1.436 (2)
C1—C2	1.396 (2)	C10—O1	1.3040 (17)
C1—N1	1.4327 (18)	C10—C11	1.417 (2)
C2—N2	1.386 (2)	C11—C12	1.351 (2)
C2—C3	1.398 (2)	C11—H11	0.9300
C3—C4	1.373 (2)	C12—C13	1.403 (2)
C3—H3	0.9300	C12—H12	0.9300
C4—C5	1.374 (3)	C13—C14	1.386 (2)
C4—H4	0.9300	C13—C15	1.424 (2)
C5—C6	1.381 (2)	C14—O3	1.3695 (17)
C5—H5	0.9300	C15—C16	1.334 (2)
C6—H6	0.9300	C15—H15	0.9300
C7—N1	1.3004 (17)	C16—C17	1.429 (3)
C7—C9	1.464 (2)	C16—H16	0.9300
C7—C8	1.490 (2)	C17—O2	1.213 (2)
C8—H8A	0.9600	C17—O3	1.3893 (17)
C8—H8B	0.9600	N1—H1	0.952 (15)
C8—H8C	0.9600	N2—H2A	0.92 (2)
C9—C14	1.4153 (19)	N2—H2B	0.91 (2)
			
C6—C1—C2	121.01 (14)	O1—C10—C9	121.51 (13)
C6—C1—N1	119.04 (14)	C11—C10—C9	119.89 (13)
C2—C1—N1	119.65 (13)	C12—C11—C10	120.84 (16)
N2—C2—C1	122.62 (14)	C12—C11—H11	119.6
N2—C2—C3	120.05 (15)	C10—C11—H11	119.6
C1—C2—C3	117.31 (14)	C11—C12—C13	121.61 (15)
C4—C3—C2	121.38 (16)	C11—C12—H12	119.2
C4—C3—H3	119.3	C13—C12—H12	119.2
C2—C3—H3	119.3	C14—C13—C12	118.17 (13)
C3—C4—C5	120.51 (15)	C14—C13—C15	118.01 (16)
C3—C4—H4	119.7	C12—C13—C15	123.82 (16)
C5—C4—H4	119.7	O3—C14—C13	119.53 (13)
C4—C5—C6	119.43 (16)	O3—C14—C9	117.17 (13)
C4—C5—H5	120.3	C13—C14—C9	123.30 (14)
C6—C5—H5	120.3	C16—C15—C13	121.81 (16)
C5—C6—C1	120.36 (16)	C16—C15—H15	119.1
C5—C6—H6	119.8	C13—C15—H15	119.1
C1—C6—H6	119.8	C15—C16—C17	120.82 (15)
N1—C7—C9	115.89 (13)	C15—C16—H16	119.6
N1—C7—C8	119.11 (13)	C17—C16—H16	119.6
C9—C7—C8	124.99 (12)	O2—C17—O3	115.12 (15)
C7—C8—H8A	109.5	O2—C17—C16	128.58 (15)
C7—C8—H8B	109.5	O3—C17—C16	116.29 (16)
H8A—C8—H8B	109.5	C7—N1—C1	126.31 (13)
C7—C8—H8C	109.5	C7—N1—H1	108.9 (11)
H8A—C8—H8C	109.5	C1—N1—H1	124.8 (11)
H8B—C8—H8C	109.5	C2—N2—H2A	118.0 (11)
C14—C9—C10	116.18 (13)	C2—N2—H2B	111.5 (13)
C14—C9—C7	123.89 (13)	H2A—N2—H2B	109.3 (17)
C10—C9—C7	119.92 (12)	C14—O3—C17	123.35 (13)
O1—C10—C11	118.60 (14)		
			
C6—C1—C2—N2	179.24 (15)	C11—C12—C13—C15	−179.15 (16)
N1—C1—C2—N2	−7.1 (2)	C12—C13—C14—O3	−178.55 (13)
C6—C1—C2—C3	1.0 (2)	C15—C13—C14—O3	1.0 (2)
N1—C1—C2—C3	174.61 (13)	C12—C13—C14—C9	0.8 (2)
N2—C2—C3—C4	−178.25 (16)	C15—C13—C14—C9	−179.68 (14)
C1—C2—C3—C4	0.0 (2)	C10—C9—C14—O3	177.82 (12)
C2—C3—C4—C5	−0.5 (3)	C7—C9—C14—O3	−1.3 (2)
C3—C4—C5—C6	0.0 (3)	C10—C9—C14—C13	−1.5 (2)
C4—C5—C6—C1	1.0 (3)	C7—C9—C14—C13	179.41 (14)
C2—C1—C6—C5	−1.5 (2)	C14—C13—C15—C16	−1.7 (2)
N1—C1—C6—C5	−175.19 (14)	C12—C13—C15—C16	177.81 (17)
N1—C7—C9—C14	174.71 (14)	C13—C15—C16—C17	−1.0 (3)
C8—C7—C9—C14	−6.3 (2)	C15—C16—C17—O2	−175.59 (18)
N1—C7—C9—C10	−4.4 (2)	C15—C16—C17—O3	4.2 (3)
C8—C7—C9—C10	174.68 (14)	C9—C7—N1—C1	−177.28 (14)
C14—C9—C10—O1	−178.93 (14)	C8—C7—N1—C1	3.6 (2)
C7—C9—C10—O1	0.2 (2)	C6—C1—N1—C7	−112.84 (17)
C14—C9—C10—C11	1.2 (2)	C2—C1—N1—C7	73.42 (19)
C7—C9—C10—C11	−179.71 (14)	C13—C14—O3—C17	2.5 (2)
O1—C10—C11—C12	179.96 (15)	C9—C14—O3—C17	−176.83 (13)
C9—C10—C11—C12	−0.1 (2)	O2—C17—O3—C14	174.78 (14)
C10—C11—C12—C13	−0.7 (3)	C16—C17—O3—C14	−5.1 (2)
C11—C12—C13—C14	0.4 (2)		

### Hydrogen-bond geometry (Å, °)

**Table:**

*D*—H···*A*	*D*—H	H···*A*	*D*···*A*	*D*—H···*A*
N1—H1···O1	0.95 (2)	1.56 (2)	2.4534 (17)	155 (2)
N2—H2A···O1^i^	0.92 (2)	2.13 (2)	2.9933 (19)	154.7 (15)
N2—H2B···O2^ii^	0.91 (2)	2.37 (2)	3.1203 (19)	138.7 (17)
C5—H5···O2^iii^	0.93	2.48	3.242 (2)	139

Symmetry codes: (i) *x*+1/2, *y*, −*z*+1/2; (ii) −*x*+3/2, *y*+1/2, *z*; (iii) −*x*+1/2, *y*+1/2, *z*.

## References

[bb1] Aazam, E. S., Fawazy, A. & Hitchcock, P. B. (2006). *Acta Cryst.* E**62**, o4285–o4287.

[bb2] Aazam, E. S., El Husseiny, A. F., Hitchcock, P. B. & Al Shehary, J. (2008). *Centr. Eur. J. Chem.* **6**, 319–323.

[bb3] Bernstein, J., Davis, R. E., Shimoni, L. & Chang, N.-L. (1995). *Angew. Chem. Int. Ed. Engl.* **34**, 1555–1573.

[bb4] El Husseiny, A. F., Aazam, E. S. & Al Shehary, J. (2008). *ICAIJ*, **3**, 64–68.

[bb5] Farrugia, L. J. (1997). *J. Appl. Cryst.* **30**, 565.

[bb6] Farrugia, L. J. (1999). *J. Appl. Cryst.* **32**, 837–838.

[bb7] Filarowski, A. (2005). *J. Phys. Org. Chem.* **18**, 686–698.

[bb8] Karabıyık, H., Ocak Iskeleli, N., Petek, H., Albayrak, Ç. & Agar, E. (2008). *J. Mol. Struct.* **873**, 130–136.

[bb9] Patil, S. A., Naik, V. H., Kulkarni, A. D. & Badami, P. S. (2010). *Spectrochimica Acta Part A*, **75**, 347-354. 10.1016/j.saa.2009.10.039 19962341

[bb10] Sheldrick, G. M. (2008). *Acta Cryst.* A**64**, 112–122. 10.1107/S0108767307043930 18156677

[bb11] Stoe & Cie (2002). *X-AREA* and *X-RED32* Stoe & Cie, Darmstadt, Germany.

